# Measures to prevent nosocomial transmissions of COVID-19 based on interpersonal contact data

**DOI:** 10.1017/S1463423621000852

**Published:** 2022-01-28

**Authors:** Tao Cheng, Jiaxing Liu, Yunzhe Liu, Xianghui Zhang, Xiaowei Gao

**Affiliations:** SpaceTimeLab, University College London, London, UK

**Keywords:** control measures, COVID-19, interpersonal contacts, medical staff, nosocomial transmission, SEIR model

## Abstract

**Background:**

With the global spreading of Coronavirus disease (COVID-19), many primary care medical workers have been infected, particularly in the early stages of this pandemic. Although extensive studies have explored the COVID-19 transmission patterns and (non-) pharmaceutical intervention to protect the general public, limited research has analysed the measures to prevent nosocomial transmission based upon detailed interpersonal contacts between medical staff and patients.

**Aim:**

This paper aims to develop and evaluate proactive prevention measures to contain the nosocomial transmission of COVID-19. The specific objectives are (1) to understand the virus transmission via interpersonal contacts among medical staff and patients; (2) to define proactive measures to reduce the risk of infection of medical staff and (3) evaluate the effectiveness of these measures to control the COVID-19 epidemic in hospitals.

**Methods:**

We observed the operation of a typical primary hospital in China to understand the interpersonal contacts among medical staff and patients. We defined effective distance as the indicator for risk of transmission. Then three proactive measures were proposed based upon the observations, including a medical staff rotation system, the establishment of a separate fever clinic and medical staff working alone. Finally, the impacts of these measures are evaluated with a modified Susceptible-Exposure-Infected-Removed model accommodating the situation of hospitals and asymptomatic and latent infection of COVID-19. The case study was conducted with the hospital observed in December 2019 and February 2020.

**Findings:**

The implementation of the medical staff rotation system has the most significant impact on containing the epidemic. The establishment of a separate fever clinic and medical staff working alone also benefits from inhibiting the epidemic outbreak. The simulation finds that if effective prevention and control measures are not taken in time, it will lead to a surge of infection cases in all asymptomatic probabilities and incubation periods.

## Introduction

The world has been experiencing an unprecedented public health emergency, a pneumonia pandemic caused by the novel severe acute respiratory syndrome coronavirus 2 (SARS-CoV-2), that is, the Coronavirus disease (COVID-19) (WHO, 2020c). This infectious disease spreads rapidly within a brief period of time to more than 190 countries or regions worldwide, resulting in acute morbidities and mortalities. As of the time of writing, there have been more than 160 million confirmed cases and over three million deaths globally (JHCRC, 2020).

The group of medical staff is a crucial point during every pandemic time (Ives *et al.*, 2009). In the context of combating the ongoing pandemic, the front-line staff build a direct relationship with infected patients (Nguyen *et al*., 2020). With the typical person-to-person transmission characteristic of COVID-19, the medical staff is more eligible to be infected or even to be an asymptomatic individual (Adams & Walls, 2020; Bai *et al*., 2020).

Many international studies and reports have pointed out that, in similar settings, the nosocomial transmissions among the medical staff are not unusual (Beijing Daily Client, 2020; Chang *et al.*, 2020; North Evening New Vision Network, 2020a; 2020b; People’s Network, 2020; Reference News, 2020; Zhou *et al.*, 2020). This is because doctors and patients in the community services centres and hospitals gather in a limited space. In emergency cases, the group gathered in the limited space is more likely to be infected with infectious diseases and cross infection (Yin, 2020).

Based on a traditional Systems Engineering Initiative for Patient Safety model, Gan *et al.* (2020) have tentatively studied the theoretical prevention to protect the health care workers from being transmitted COVID-19 in the workplace. Harrison *et al*. (2020) have stated that the infectious possibilities within the hospital are highly possible with 2.5% proportions to the whole infected cases by the data of independent search from current works of literature, while Long *et al*. (2020) have showed that typically the asymptomatic cases are common and have higher virus levels. Studies have also shown that different types of medical personnel may contribute to different degrees of infectious possibilities. Particularly, if the staff has received the pre-training course on self-protection, they could have a bit lower infected rates (*P* < 0.05) (Zhou *et al.*, 2020).

It is evident from the literature that the COVID-19 has infected a considerable number of medical personnel through nosocomial transmission. It is also noticeable that the infection of medical workers mainly occurred in the early stage of the epidemic when anti-epidemic measures and policies were not introduced. Recent evidence has approved that subclinical patients (i.e., infected persons with pre-symptomatic or asymptomatic cases of COVID-19) can be contagious and transmit the virus efficiently (Chang *et al*., 2020; WHO, 2020a). These findings suggest that it is insufficient that only adopt the conventional personal measures of protection to ensure the safety of the medical staff during this ongoing pandemic, such as wearing regular face masks, goggles and protective gowns. Given that medical staff may also be a dangerous transmitter among the whole pandemic to their family or community, proactive measures should be taken to reduce the nosocomial transmission among staff members.

Although there are many epidemic studies on COVID-19 spread patterns and the infection of medical staff (Fauver *et al*., 2020; Kamel Boulos & Geraghty, 2020; Prem *et al*., 2020; Sotgiu *et al*., 2020; Zu *et al*., 2020), few studies have conducted detailed spatiotemporal analysis regarding the virus spread dynamics within the hospital with the interpersonal data so that the effective control measures could be implemented in practice. This paper addresses this gap by analysing the interpersonal data and the outbreak situation in hospitals so that effective non-pharmaceutical measures and policies could be identified to prevent the nosocomial transmissions of COVID-19.

The paper is organised as follows. The next section introduces the interpersonal data used for the case study in detail, describing the process of data acquisition of medical staff during the pandemic period. The third section presents the methodology. It first defines the concept and method of effective distance which is developed to quantify the transmission rate based upon interpersonal contacts among individuals in the hospital. It then revises a Susceptible-Exposure-Infected-Removed (SEIR) model to accommodate the hospital situation with in and out population, and the asymptomatic and latent infections of COVID-19. The proactive control measures are drawn from the observation of the operation of the case study hospital to minimise the contacts among individuals. The fourth section demonstrates the influence of the proactive control measures on epidemic transmission using the revised SEIR model. In particular, it discusses the impacts of the asymptomatic and latent infection in the epidemic situation. The fifth section summarises the major findings with policy suggestions, in consistency to the latest (WHO, 2020b) on protecting the safety of medical staff. This section also describes the limitations and future work of our research.

### Data description

The interpersonal contact data were extracted from a Primary general hospital located in a medium size in Hebei province, China. According to the scale of the hospital and its ability to provide medical care, hospitals in China are organised by a three-tier hierarchical system, comprising *Primary*, *Secondary*, and *Tertiary* institutions (Li *et al*., 2008). Most hospitals in towns are classified as *Primary*, which is the fundamental choice for most residents to diagnose their medical condition without an emergency. In 2018, there were nearly 8000 *Primary* general hospitals in China, accounting for 40.55% of the total number of general hospitals. Given the wide geographical distribution and the large number of hospitals, the selection of such a *Primary* hospital is accordingly representative for China.

The interpersonal contact data were derived from a field survey and the internal recording system of the hospital in December 2019 and February 2020, which captured the contacts between medical staff and patients in both inpatient and outpatient departments, without considering interpersonal contacts among admin staff. After interviewing staff members, we noticed that the staff from different medical departments contact each other two to five times per day. The specific data collection processes are summarised as follows:

- Inpatient Department: the daily contact times between nurses and inpatients and the times of doctor rounds (which can be regarded as the contact times with nurses and patients) are highly regular. This is because the number of times nurses care for inpatients is fixed unless there is an emergency, but this kind of contingency will not affect the results of the study. Therefore, through field investigation, the number of daily interpersonal contacts of medical staff in the inpatient department can be obtained (Table 1).
  **Table 1.** Interpersonal contact data in the inpatient department in December

Type of contact	Number
Between nurses and patients	9
Nurse 1	2
Nurse 2	3
Nurse 3	4
Between doctors and patients	8
Doctor 1	3
Doctor 2	3
Doctor 3	2
Between doctors and nurses	30
Doctor 1 and Nurse 1	4
Doctor 1 and Nurse 2	5
Doctor 1 and Nurse 3	5
Doctor 2 and Nurse 1	4
Doctor 2 and Nurse 2	6
Doctor 2 and Nurse 3	6

For outpatient, they are four departments that interact with patients as follows:

- Internal Medicine Clinic: there are 10 medical staff members working in the same office. After investigation, we found that the doctor would contact each patient twice in a consultation. This is because the doctor will first examine the patient then meet the same patient after laboratory testing. Therefore, the medical outpatient’s visit data multiplied by two represent the daily contact times of the medical outpatient doctor with the patients.
- Surgical Clinic: There are five medical staff members in the same office. Similar to the medical clinic, a patient needs to contact the surgical doctor twice in a consultation. Therefore, the number of times the medical staff in the surgical department contact the patient can be calculated from multiplying the number of consultations by 2.
- Laboratory: There are eight staff members in the laboratory section of the hospital. They are separated into two offices, respectively in charge of blood tests and routine urine tests. Every time a patient comes for a test, s/he will contact all the staff members in one test office. Therefore, every patient test here is recorded as one contact with all the staff members in the office of the test.
- Pharmacy: There are seven staff members in the hospital pharmacy. According to the duty schedule, while the four staff members are working, the rest of three are resting, plus one staff member is rotated every day. Therefore, four pharmacy members work in the pharmacy at the same time, so each patient will contact each staff member when taking medicine.

The interpersonal contact data between medical staff members with the outpatients are shown in Tables 2 and 3, in December 2019 and February 2020, respectively.

**Table 2.** Example of contact data between outpatient medical staff and patients in December

	1-Dec	2-Dec	3-Dec	4-Dec	5-Dec	6-Dec	7-Dec	……	30-Dec	31-Dec
Internal medicine	76	312	296	284	264	296	68	……	360	412
Doctor 1	0	22	18	20	22	22	0	……	20	24
Nurse 1	0	32	34	34	38	36	0	……	32	34
Surgical clinic	0	124	140	132	120	112	0	……	0	124
Doctor 1	0	18	20	24	18	14	0	……	0	18
Nurse 1	0	2	2	2	2	2	0	……	2	2
Laboratory	0	9	11	8	10	18	0	……	3	
Doctor 1 (routine blood test)	0	6	9	3	8	12	0	……	3	
Doctor 2 (biochemistry)	0	2	2	3	2	4	0	……	0	
Pharmacy	48	77	83	91	75	110	66	……	0	0
Pharmacist 1	48	77	0	0	75	110	66	……	0	0
Pharmacist 2	48	77	83	0	0	110	66	……	0	0

**Table 3.** Example of contact data between outpatient medical staff and patients in February

	1-Feb	2-Feb	3-Feb	4-Feb	5-Feb	6-Feb	7-Feb	……	28-Feb	29-Feb
Internal medicine	32	36	108	100	108	96	104	……	92	20
Doctor 1	0	0	14	10	16	12	10	……	12	0
Nurse 1	4	4	4	2	4	4	4	……	12	0
Surgical clinic	16	16	18	18	16	18	16	……	18	16
Doctor 1	0	0	22	24	26	28	44	……	18	0
Nurse 1	0	0	2	0	0	0	0	……	2	0
Laboratory	3	5	5	4	3	2	4	……	5	8
Doctor 1 (routine blood test)	2	3	5	3	1	1	4	……	3	4
Doctor 2 (biochemistry)	0	0	0	0	0	0	0	……	0	2
Pharmacy	77	29	76	55	44	54	52	……	83	40
Pharmacist 1	77	0	0	55	44	54	52	……	83	40
Pharmacist 2	77	29	0	0	44	54	52	……	0	40

**Figure 1.**
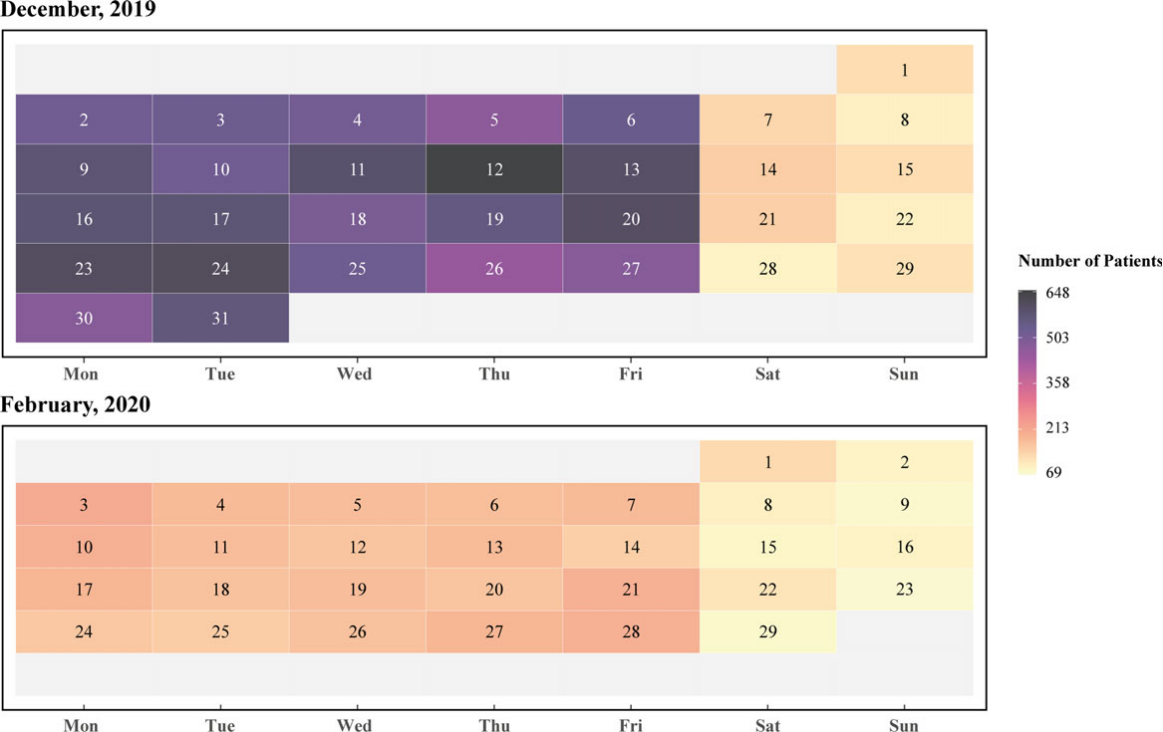
Comparison of the number of patients in the hospital before (i.e., December 2019) and after (i.e., February 2020) the COVID-19 outbreak.

Figure 1 contains daily patients’ data in December 2019 and February 2020. The higher number in December shows that the hospital operated normally in December because the outbreak has not yet exploded. After the pandemic was officially reported in late January, the number of patients decreased considerably.

## Methodology

Based upon the scenarios of the hospital operation presented in the “Data description” section, we developed three steps to conduct the research. First, we define the rate of virus transmission between individuals based upon their interpersonal contacts in the hospital. Then the possible prevention measures are proposed to minimise the contacts between individuals, in order to reduce the transmission. Finally, an improved SEIR model is proposed to simulate the effectiveness of the prevention and control measures. This improved SEIR model is modified to accommodate the hospital situations and the asymptotic and latency specific to COVID-19.

### Rate of transmission based on effective distance

Since COVID-19 is mainly spread from person to person, the nosocomial transmission somehow depends on the distance or frequency between patients and the medical staff members. Effective distance has been proposed to describe the chance of disease transmission between individuals (Deutsch & Isard, 2007). The effectiveness of this measurement method has been verified on the actual data of H1N1 and SARS-CoV-2 transmission (Asai & Nishiura, 2018; Shi *et al*., 2020). Therefore, we use effective distance to measure the chance of COVID-19 transmission between patients with medical staff.

The equation (Eq. 1) of effective distance is defined as below:
(1)

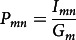

where 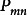 represents the contact probability between two individuals *m* and *n*. *I*_*m*n_ represents the number of contacts between two individuals *m* and *n* in the hospital setting. 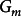 represents the sum of the number of contacts between the individual *m* and all the other individuals.

Because the logarithm is additive, the logarithm of the obtained contact probability 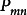 is taken. Finally, the effective distance 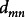 is obtained as follows (Eq. 2):
(2)

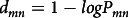




### Protective measures for medical staff

COVID-19 virus is more likely to spread among the two individuals who have frequent contacts and long contact. Given that we do not have the information of the time of the contact, we focus on the contact frequencies. To reduce the speed and scale of virus transmission, it is necessary to reduce the number of contacts between individuals in hospitals. Although other measures could be implemented, here we propose the followings given these measures can keep the hospital running at maximal capacity:

- Medical staff works alone: When the number of symptomatic individuals in the hospital reaches a fixed threshold, establish a separate working space in the hospital, let the medical staff abide by the policy, stick to their posts, work independently and reduce contact with each other.
- Establishment of a separate fever clinic: When the number of symptomatic individuals in the hospital reaches a fixed threshold, a separate fever clinic was established to treat patients with fever symptoms. The fever clinic was completely isolated from other outpatient departments.
- Adopt a rotation system of medical staff: When the number of symptomatic individuals in the hospital reaches a fixed threshold, the medical staff are divided into two groups. Each group worked continuously for 14 days and was isolated for 14 days. During the 14 days of isolation, the other group stuck to their posts.

All of the measures described above depend on one parameter, that is, the number of people with symptoms in the hospital reaches a fixed threshold, and the trigger threshold of the measures and policies considered in this paper is 1. For example, if the threshold value is 1, when the number of symptomatic individuals in any department or patient is greater than 1, the above measures and policies will be adopted.

Because the hospital is different from other infrastructure, it cannot be closed during the epidemic period, so patients will come to see a doctor and consult every day. According to all the prevention and control measures and policies, there are patients entering the hospital every day, and there are also patients discharged from the inpatient department after rehabilitation.

### The modified SEIR model

Susceptible-Infected-Removed (SIR) model has been widely used for prediction and the estimation of epidemiological parameters. SEIR model is an extension to SIR by adding exposed population, which has been widely used in the modelling of SARS, Ebola and other epidemics (Dye & Gay, 2003; Rachah & Torres, 2017). The SEIR model has excellent performance for characterising the epidemic dynamic and predicting possible contagion scenarios. In the recent studies on the COVID-19 pandemic, SEIR and its derivative model have been widely employed to assess the effectiveness of various non-pharmaceutical measures, such as lock-down and quarantine (Yi *et al.*, 2009), predict epidemic evolution (Yang *et al*., 2020) and evaluate management strategies (R&acaron;dulescu *et al.*, 2020) by combining with some statistical (Pandey *et al.*, 2020), AI (Yang *et al*., 2020) and simulation models (Annas *et al.*, 2020).

Yang *et al.* (2020) have accurately predicted that the COVID-19 epidemic in China will reach its peak in late February 2020 and tend to be flat at the end of April using a modified SEIR model. They revised the SEIR model to accommodate the dynamic population of a province to simulate the transmission in China by adding immigrants and emigrants to the susceptible population. Inspired by their work, here we further improve their SEIR model as follows to accommodate the situation in hospitals:

- In and Outpatients: Because the number of patients who come to the hospital every day is not fixed, and there are patients discharged every day, it is necessary to add the number of daily visits and discharge into the model to dynamically show the changing state of susceptible population [S]. Therefore, we divide the changing susceptible population of the hospital as In (visitor) and Out (leave), represented as 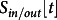 . So are the numbers of the exposed population, represented as 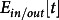 .
- Latency: We separate the number of latent and sick persons with different probabilities of infecting susceptible persons and exposed persons, which will be used to simulate the epidemic prevention situation in the hospital after the introduction of epidemic prevention measures (the sick will be isolated).
- Asymptomatic: Exposed population (E) could become symptomatic or asymptomatic (A) patients, with rates, 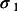 and 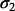 , respectively. Symptomatic patients will be classified as infected (I) and will be isolated without further infection to others. We separate the asymptomatic patients from the (I) due to the fact that asymptomatic patients are not easily identifiable who will keep on infecting others (given they show no symptom) until they recover. This is different from Yang *et al*. (2020).
- Incubation: Furthermore, the incubation period will be added to the model with a median of 7 days in latency as analysed by Dietz (1992). If the incubation period is infectious, the asymptomatic population (A) will continue to affect the exposed (E) population with a rate 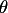 .

**Figure 2.**
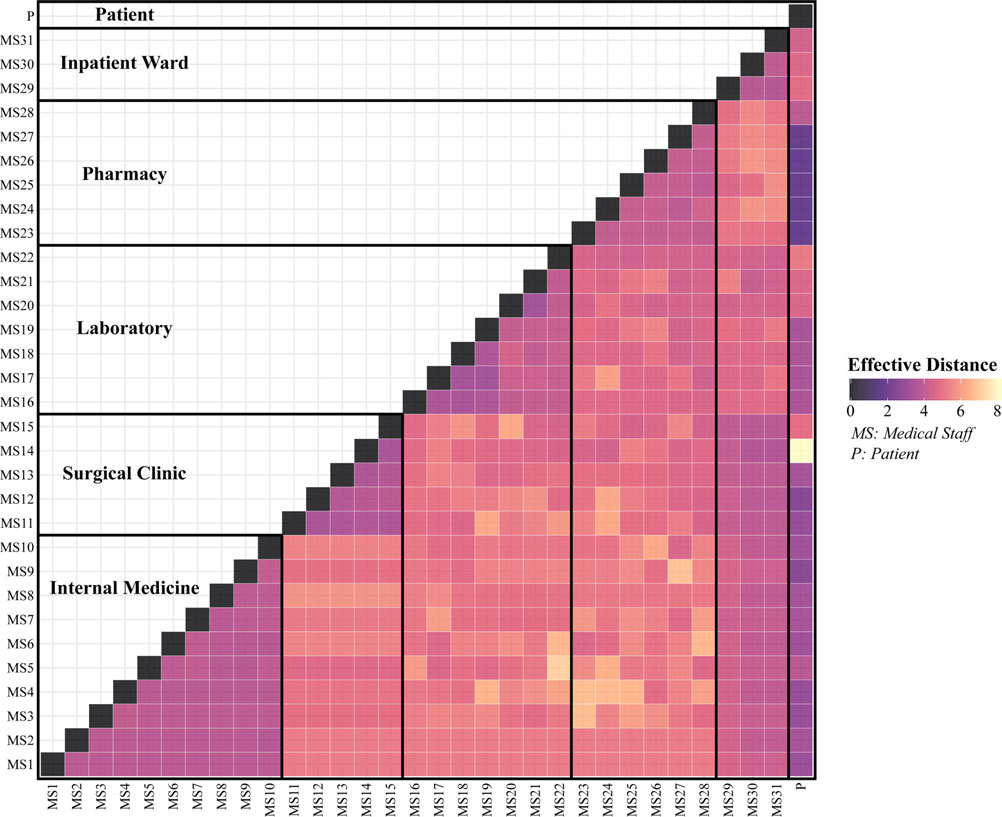
Improved SEIR model

Our improved SEIR model is shown in Figure 2.

The formula of the improved SEIR model is as follows:
(3)

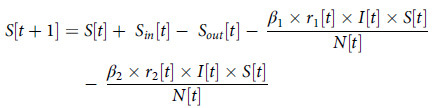



(4)

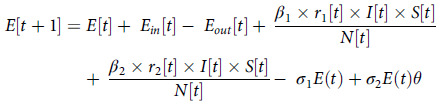



(5)

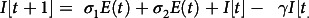



(6)

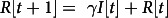



(7)

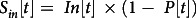



(8)

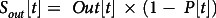



(9)

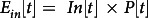



(10)

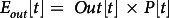

where 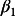 is *the* infection rate of the susceptible population; 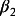 is the infection rate of the susceptible population by exposed persons; *r*_1_ is the daily average number of people exposed to infection and *r*_2_ is the average number of exposed people; *P*[t] is the probability of exposure among In and Out population, which is calculated based upon the effective distance as defined above; 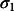 is the probability that exposed population convert into symptomatic infection; 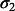 is the probability that the exposed population convert into asymptomatic infection; 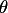 is the probability that asymptomatic infection converts into the exposed group. 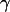 is the probability of infected convert into recovery (R).

In order to apply the SEIR model, it is necessary to estimate the parameter *β*, which is the product of the number of daily contacts (*r*) and the probability of infection (*b*). According to the data collected in the hospital in our case, the average daily contact number of infected persons is consistent with that of exposed persons, that is, *r*_1_ = *r*_2_ = 14. According to the literature (Yang *et al*., 2020), *b* = 0.0529. Therefore, 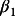 = 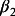 = *b* * *r* = 0.7406 in our case study when no control measures are implemented (see the “Effective distance of medical staff” section). However, when different response measures are taken, the number of daily contacts corresponding to different groups will change in varying degrees, as the embodiment of the measures taken (see “Influence of three prevention and control measures on hospital epidemic” section).

## Results

### Effective distance of medical staff

**Figure 3.**
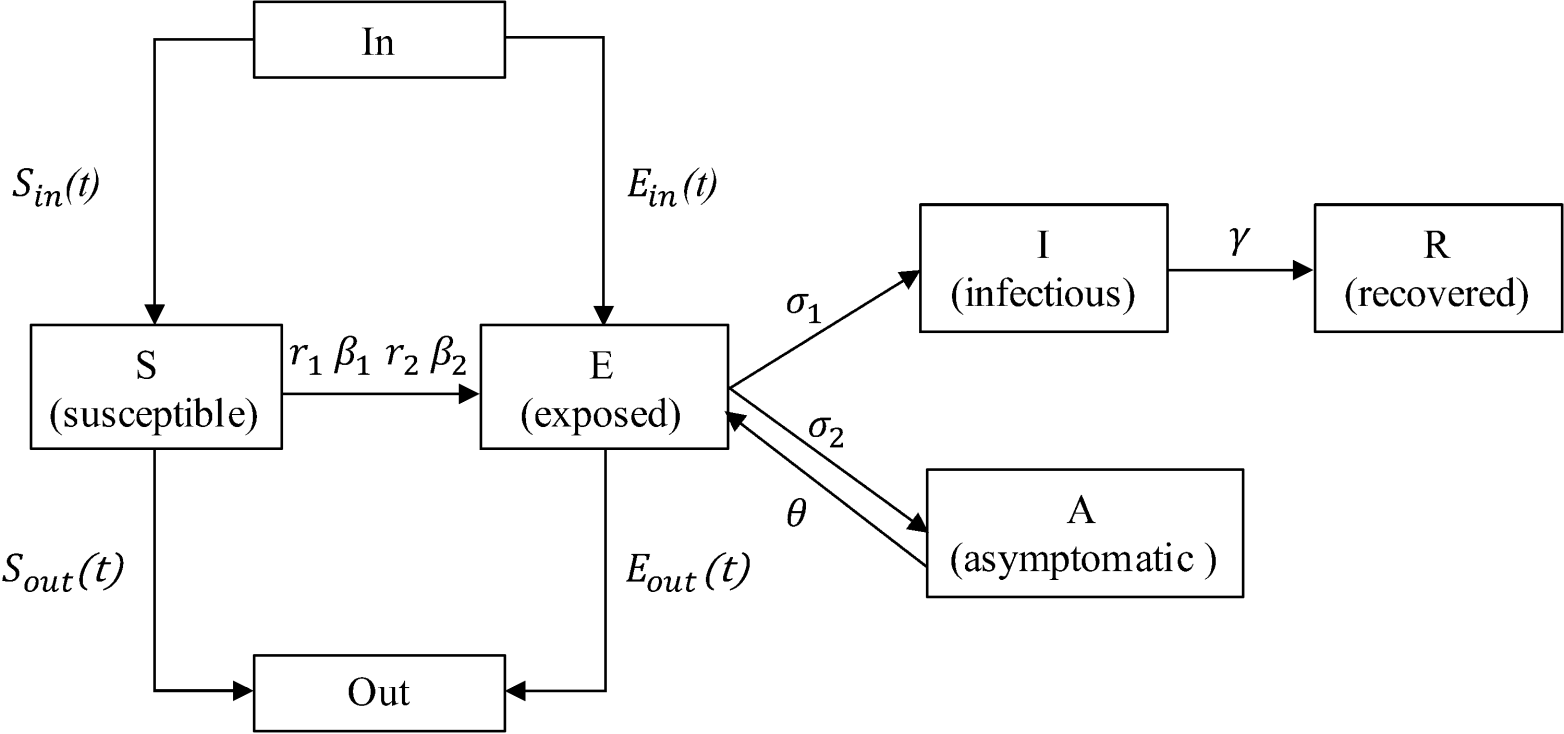
Calculated effective distance between individuals in the hospital

Based upon the interpersonal contact data collected in Tables 1–3, the effective distance 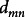 among the medical staff members and the patients is calculated as a pairwise distance matrix D. A heatmap is generated based on the matrix D (Figure 3), showing the pairwise effective distance between all medical staff. In Figure 3, the medical staff were categorised based on their department, and the patients are regarded as a whole and placed in the last part of the matrix as this paper is intended to solve the safety problem of medical staff.

As can be seen from Figure 3, the effective distance of medical staff in the internal medicine outpatient department, surgical outpatient department, laboratory, pharmacy and inpatient department is relatively short, which indicates that the internal staff of the department have more contact times; although the laboratory is divided into two offices of blood routine and urine test, the effective distance between the two offices is also short, indicating that the personal contact between the offices is also more frequent. In contact with the patients, the number of pharmacy staff is more, followed by internal medicine and surgical outpatient. Because of the short effective distance between the outpatient department and the medical ward, the medical staff often needs to check. Generally, the greater the effective distance between the different departments, the fewer connections they have and lower rate of transmission.

### Influence of three prevention and control measures on hospital epidemic

Here we present the simulation results using the improved SEIR model to evaluate the effectiveness of the protective measures for medical staff proposed in the “Protective measures for medical staff” section, including *medical staff works alone*, *establishment of a separate fever clinic* and *adopt a rotation system of medical staff*. The impacts of asymptomatic infected persons (A) will be further explored in the “Influence of asymptomatic probability on epidemic transmission”. The “Influence of latent period infectivity on epidemic transmission” section investigates the impact of the latent period infectivity on epidemic transmission.

**Figure 4.**
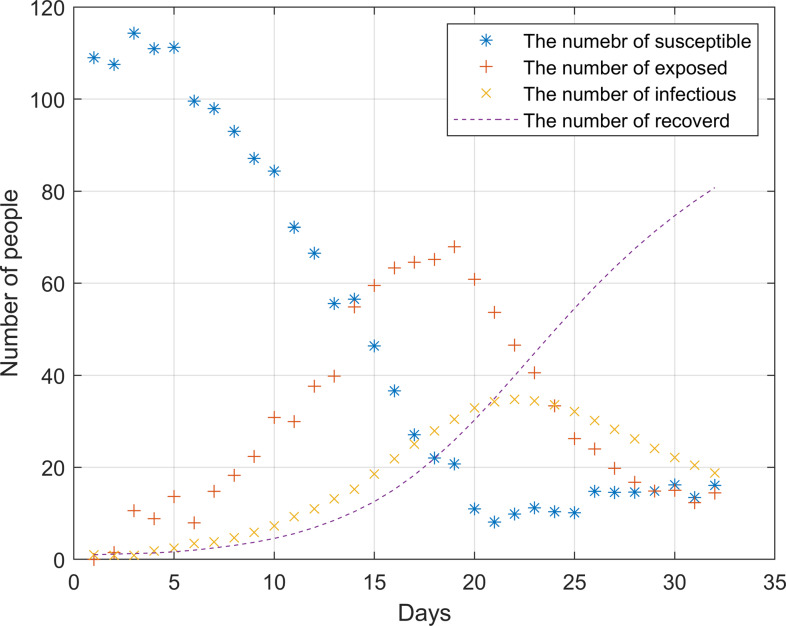
Simulation of hospital outbreak without any measures and policies

Since the outbreak of the epidemic has not yet occurred in December 2019, the model based on the data at this time can better reflect the real situation of the outbreak in the hospital. Therefore, the hospital contact data and admission data in December 2019 are selected for simulation. In the analysis, the change of infected individuals and the median of latecomers were considered in the analysis. Day 0 represents the first day. Figure 4 shows the outbreak of the epidemic in the hospital from 0 to 32 days without any prevention and control measures and policies.

After the outbreak of the epidemic, the number of susceptible persons dropped sharply before the 20th day. During this period, the infection rate was significantly higher than the cure rate. On the 22nd, the epidemic broke out to the highest point, about 35 people were ill. As there were almost no susceptible individuals on the 20th, the number of infected individuals began to decline. The number of infected and cured patients was the same around the 21st day, it was not until the 25th that the epidemic was brought under control.

**Figure 5.**
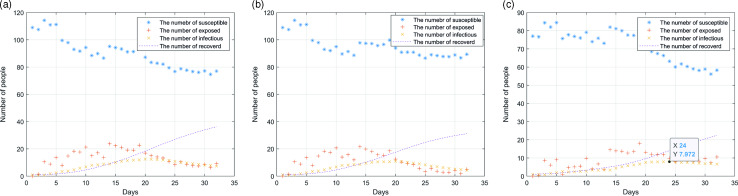
Simulation of the outbreak in the hospital after the implementation of relevant measures: (a) medical staff works alone; (b) establishment of a separate fever clinic; (c) adopt rotation system of medical staff.

Figure 5a, 5b and 5c, respectively, shows the impact of three measures and policies on the epidemic situation, including allowing medical staff to work alone, establishing a separate fever clinic and applying the rotation system of medical staff. These diagrams demonstrate that the three measures can effectively prevent and control disease transmission. Among them, the medical staff working alone and establishing a separate fever clinic can effectively control the whole process. The peak number of infected people is less than 15, and the days reaching the peak are about 23 days. The medical staff rotation system, however, is most effective for the epidemic prevention and control, with eight infected persons as the peak number.

After analysis, it can be found that this is because the rotation system greatly reduces the probability of mutual contact between medical staff. Although the workload of the staff will increase, they can get 14 days’ rest if they stick to it for 14 days. Also, it can be found in the analysis chart that the number of infected people will rebound after taking these three measures. This is because more people are coming to the hospital on that day, which increases the probability of personnel contact. Therefore, the hospital should control the number of patients during the epidemic period. Once the patients in the hospital are saturated, the flow of people should be diverted to other hospitals to reduce the pressure.

### Influence of asymptomatic probability on epidemic transmission

The purpose of establishing a separate fever clinic is to isolate the infection and suspected cases from other patients who come to the hospital, as well as the staff of other departments of the hospital, to achieve the purpose of prevention and control. However, how the proportion of asymptomatic infection affects the spread of the epidemic in other departments of the hospital is an important issue worthy of attention. At present, there is no consensus on the proportion of asymptomatic patients. Therefore, multiple asymptomatic probabilities are employed here to simulate its impacts.

**Figure 6.**
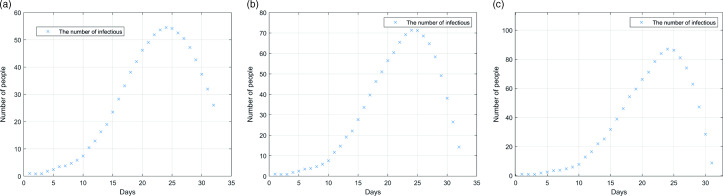
Simulation of different asymptomatic probabilities of σ_2_: (a) σ_2_ = 0.1; (b) σ_2_ = 0.2; (c) σ_2_ = 0.3.

Figure 6 shows the epidemic situation without prevention and control measures, with σ_2_ of 0.1, 0.2 and 0.3, respectively. It can be found that the epidemic situation corresponding to different σ_2_ values is significantly different. With the increase of σ_2_, the rate of case increase is faster. When σ_2_ = 0.1, the peak of the number of cases will reach 55 at most, and the number of cases will begin to decrease after 24 days. With the increase of σ_2_, the scale of infection increased significantly, and the infection rate was accelerated. When σ_2_ is 0.2, the peak number of infected people is 71, while when σ_2_ is 0.3, the number of infected people will be close to 90.

**Figure 7.**
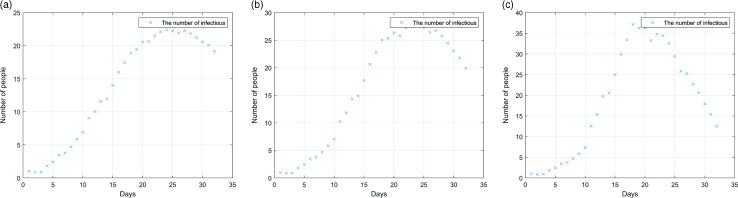
Influence of prevention and control measures with different asymptomatic probability: (a) σ_2_ = 0.1; (b) σ_2_ = 0.2; (c) σ_2_ = 0.3.

Figure 7 shows the new epidemic situation under the control measures. The prevention and control measures were defined as the establishment of a separate fever clinic (threshold = 1) and simulated under different asymptomatic probabilities. It can be found that after the implementation of prevention and control measures, the outbreak of the epidemic has been controlled to a certain extent, especially when σ_2_ is 0.1, the peak number of infected people is reduced from 55 to 23, and even if σ_2_ is 0.3, the peak number of infected people will not exceed 40. And the peak of the epidemic has been prolonged, which can reduce the pressure on the hospital and arrange the epidemic prevention materials more reasonably.

### Influence of latent period infectivity on epidemic transmission

The novel coronavirus pneumonia will spread and hide in the incubation period. This phenomenon brings great difficulties in epidemic prevention and control. At present, novel coronavirus pneumonia can only be identified as infectious in the incubation period, but the accurate transmission capacity and the infection days in the incubation period are not yet determined. In order to compare the influence of latent period infectivity on the spread of epidemic situations, this paper simulated three situations: *non-infectious in the incubation period*, *infectious in the last day of the incubation period* and *infectious in the last two days of the incubation period*.

**Figure 8.**
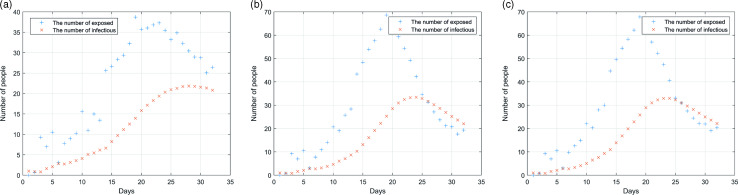
Influence of latent infectivity on epidemic situation: (a) the incubation period was not infectious; (b) infectious on the last day of incubation; (c) the last 2 days of incubation period are infectious.

Figure 8 shows the change of the median of individual cases in the simulation over time in these three cases and shows the situation of hospital outbreak without prevention and control measures (threshold is 1).

As can be seen from Figure 8, compared with the other two cases, when there is no infection in the incubation period, the transmission speed of the epidemic is the slowest, and the peak number of cases is also the lowest. When the incubation period is infectious, no matter how many days of infection, the number of cases will reach the peak around 20 days, and the transmission speed will accelerate with the increase of infection days. It should be noted that in these three cases, the number of infected people accounted for more than 30% of the hospital medical staff, that is to say, no matter what the situation of transmission in the incubation period, if no prevention and control measures are taken, there is a high probability of outbreak.

## Conclusions and discussion

This research employed micro-scale interpersonal contact data to analyse and simulate the impacts of various prevention and control measures to contain the virus transmission in hospitals and further explore the influence of asymptomatic infection and incubation periods. This research presented four key findings.

First, the effective distances between the medical staff show that the staff members in outpatient departments have more contact with patients, especially those working in the pharmacy. It should be noted that the number of inpatient nurses contacting patients is far less than the number of outpatient staff contacting patients. However, the doctors in the outpatient department need to connect with the inpatient department and make rounds, so they have more contact with the medical staff and patients in the inpatient department. The results show that the virus is more easily transmitted within the hospital.

Second, three proactive control measures are proposed to minimise the personal contacts within the hospital, including *medical staff works alone*, *establishment of a separate fever clinic* and *a rotation system of medical staff,* once there is one patient with COVID-19 symptoms in any departments of the hospital. This will effectively reduce the contacts among doctors and between medical staff with the patents.

Third, an improved SEIR model was adopted here to simulate the transmission of the epidemics within the hospital to evaluate the effectiveness of the control measures. The simulation shows that the virus will spread rapidly and cause large-scale infection in the hospital before adopting control measures. After applying different prevention and control measures, the transmission speed of COVID-19 and the cumulative number of infected people decreased significantly. In the whole epidemic cycle, the implementation of the medical staff rotation system has the most significant impact on the prevention and control of the epidemic. Besides, the establishment of a separate fever clinic and medical staff working alone also benefits from inhibiting the outbreak of the epidemic.

Last, asymptomatic patients and patients within incubation periods bring new challenges to contain COVID-19 transmission. The simulation finds that if effective prevention and control measures are not taken in time, it will lead to a surge of infection cases in all asymptomatic probabilities and incubation periods.

These findings contribute a body of evidence on formulating reasonable intervention policy and containing the virus transmission. These analysis results and policy suggestions are also consistent with the recent WHO initiative to protect the safety of medical staff (WHO, 2020b). Community Service Centres and hospitals need to make timely responses and take effective measures, such as purchasing sufficient medical materials, optimising the consultation processes and strengthening daily management.

However, our analysis presents the following limitations, which in turn reveal avenues for future research. First, COVID-19 is a novel virus, and human beings know little about it. With the deepening of people’s understanding of COVID-19, there is still space for improving the model parameters. Second, the interpersonal contact data are collected from regular records and the hospital database. The dataset ignored the random activities and the contacts among admin staffs, which may affect the accuracy of results. Future studies should incorporate other data sources to improve the collection completeness of interpersonal contacts. For instance, the movement of the patients within the hospital, and the possible contact they might have with each other. Moreover, the length of contact can also be considered in defining the effective distance. Also, collecting the real infection data of medical staff and patients in the early stage of the pandemic will benefit from improving the accuracy of results and conducting the comparative analysis.

## Acknowledgements

This work was supported by the UKRI Medical Research Council: COVID-19 Rapid Response: Virus Watch: Understanding Community incidence, Symptom profiles, and Transmission of COVID-19 in relation to Population Movement and Behaviour (MC_PC_19070). The authors acknowledge the hospital for the provision of the contact data. The authors would like to thank the anonymous reviewers for their comments.
